# Pseudorabies virus IE180 protein hijacks G3BPs into the nucleus to inhibit stress granule formation

**DOI:** 10.1128/jvi.02088-24

**Published:** 2025-03-27

**Authors:** Ruihan Zhao, Zhenbang Zhu, Wenqiang Wang, Wei Wen, Zhendong Zhang, Herman W. Favoreel, Xiangdong Li

**Affiliations:** 1 Jiangsu Co-innovation Center for Prevention and Control of Important Animal Infectious Diseases and Zoonoses, College of Veterinary Medicine, Yangzhou University College of Veterinary Medicine 614704, Yangzhou, China; 2 Department of Translational Physiology, Infectiology and Public Health, Faculty of Veterinary Medicine, Ghent University 366759 , Merelbeke, Belgium; 3 Joint International Research Laboratory of Agriculture and Agri-Product Safety, the Ministry of Education of China, Yangzhou University 38043, Yangzhou, China; University of Toronto, Toronto, Ontario, Canada

**Keywords:** PRV IE180, stress granules, G3BP1/2, nuclear localization

## Abstract

Pseudorabies virus (PRV) is a porcine alphaherpesvirus that can infect different animal species and cause pruritus and lethal encephalitis. Stress granules (SGs) are membrane-free cytoplasmic structures formed by liquid-liquid phase separation of G3BP proteins during cell translation inhibition, which generally plays an antiviral role in various viral infections. In this study, we found that infection with different PRV strains inhibits the formation of SGs in host cells. We found that IE180, the only immediate early protein of PRV, has a distinct inhibitory effect on SG formation and colocalizes with SG-nucleating G3BP proteins (G3BP1/2) in the nucleus during PRV infection. Co-immunoprecipitation assays demonstrated an interaction between IE180 and G3BP1/G3BP2, and this interaction appears to depend on the Herpesvirus ICP4-like protein N-terminal (ICP4L-N) domain of IE180 and both NTF2L and RBD domains of G3BP1. Since G3BPs mainly function in the cytoplasm to induce SG formation, we constructed several IE180 protein truncations lacking a nuclear localization sequence to alter the subcellular localization of IE180 to the cytoplasm. Mutant IE180 protein was mainly expressed in the cytoplasm and still suppressed SG formation induced by arsenite or poly(I:C), but its ability to inhibit SG formation was weakened. Importantly, knockout of G3BPs facilitated PRV replication in H1299 cells, while exogenous expression of G3BPs and formation of SGs in wild-type H1299 cells suppressed PRV replication. In summary, our study indicates that PRV IE180 suppresses SG formation and hijacks G3BPs into the nucleus to benefit virus replication.

**IMPORTANCE:**

Herpesviruses, including pseudorabies virus (PRV), have evolved different strategies to compromise host immune responses. Stress granules (SGs) are one of the targets that viruses can overcome in order to increase replication. The related herpes simplex virus 1 (HSV-1) inhibits SG formation to promote virus replication, but the underlying mechanisms remain unknown. In this study, we confirmed that infection with different PRV strains inhibits SG formation. Interestingly, we found that the PRV immediate early protein IE180 interacts with G3BP proteins and hijacks these proteins into the nucleus to prevent SG formation. In line with the antiviral effect of SGs on PRV replication, exogenous expression of G3BPs and formation of SGs in G3BP1/2 knockout H1299 cells significantly compromised PRV replication. The reported mechanism appears to be also utilized by HSV-1 to prevent SG formation. Therefore, our study elucidates a novel mechanism by which alphaherpesviruses inhibit SG formation, which provides a new perspective to inquire into the immune escape of PRV and other alphaherpesviruses.

## INTRODUCTION

Pseudorabies (PR) is an acute infectious disease caused by pseudorabies virus (PRV, suid alphaherpesvirus 1, and Aujeszky’s disease virus). Clinical manifestations in PRV-infected pigs include reproductive disorders in gestating sows, testicular inflammation in boars, acute mortality in newborn piglets, and respiratory symptoms and growth retardation in fattening pigs, posing a serious hazard to the global pig industry (1). PRV belongs to the Herpesviridae family, with a double-stranded linear DNA genome of about 150 kb. Viral gene expression in PRV is similar to that in herpes simplex virus 1 (HSV-1), both belonging to the alphaherpesvirus subfamily. The replication of PRV is tightly regulated, and the immediate-early genes, early genes, and late genes are expressed in sequence (2). Although PRV epidemics have been effectively controlled by the use of different vaccines including BarthaK61, Ea, and Fa, the emergence of PRV mutants, particularly in Asia, has posed a challenge to prevent and control PRV in recent years (3). Recently, a PRV variant strain named hSD-1/2019 was isolated from a human patient with acute encephalitis, which provided the first direct evidence of rare human infection with PRV (4). Since then, at least seven presumed human cases of PRV infection have been reported, and the cardinal symptoms of patients include endophthalmitis and encephalitis (4–10).

When mammalian cells are subjected to a variety of environmental stresses (including viral infection), they maintain a relative homeostasis by temporarily inhibiting cellular protein translation (11, 12). Stress granules (SGs) are subsequently formed in the cytoplasm to assemble cytoplasmic mRNAs, protein translation factors, and RNA-binding proteins in response to different kinds of stressors (13). The general pathway of SG formation occurs via phosphorylation of eukaryotic translation initiation factor eIF2α by either of the eIF2α kinases Protein Kinase R (PKR), Protein kinase R (PKR)-like endoplasmic reticulum kinase (PERK), General control nonderepressible 2 (GCN2), or Heme-regulated kinase (HRI) (14). When the cell protein translation is blocked by phosphorylated eIF2α (or eIF4A in an eIF2α-independent pathway), specific SG nucleation factors contribute to SG assembly, such as Ras-GTPase-activating protein-binding proteins (G3BP) and T-cell intracellular antigen 1 (TIA1) (15). Among these, G3BP proteins are pivotal in driving SG nucleation (16). Subsequently, a liquid-like layer is formed around the core by liquid-liquid phase separation (LLPS), resulting in the formation of the SG, which plays an indispensable role in host resistance to viral infection (17, 18).

To maximize their replication efficiency and counter host defenses, viruses may hijack the host’s translation system to disrupt SG aggregation and promote their replication (19, 20). In this process, viruses usually target G3BPs to block SG formation directly. For example, porcine epidemic diarrhea virus (PEDV) triggers the induction of SGs early in infection but triggers cleavage of G3BP1 via caspase 8 to inhibit their formation late in infection (21). The African swine fever virus S273R protein directly cleaves G3BP1 as a protein hydrolase (22). Meanwhile, viruses also inhibit SG formation through indirect pathways. For example, Sendai virus promotes viral replication by inhibiting the phosphorylation of eIF2α and suppressing SG formation (23). Vesicular stomatitis virus (VSV) infection degrades nuclear factor 90 (NF90) by up-regulating T cell immunoglobulin mucin-3 (Tim-3) in macrophages, further blocking SG formation induced by PKR activation (24).

IE180 is the only immediate early protein of PRV, which is homologous to HSV-1 ICP4 (25). Immediate early proteins of herpesviruses are generally related to activation of viral gene transcription and regulation of the host’s antiviral defense (2, 26, 27). Although several previous reports have explored some of the functions of IE180, including its role in transactivation of viral genes and promoting virus replication (28–30) and its ability to inhibit eIF2α phosphorylation (31), a possible role of IE180 in SG suppression and immune escape during PRV infection remains unclear. In this study, we investigated PRV-mediated inhibition of SG formation and found that PERK activation was inhibited during the early stages of PRV infection. Moreover, we found that the IE180 protein of PRV interacts with the NTF2L and the RGG/RRM structural domains of G3BP proteins to inhibit SG formation by hijacking G3BP into the nucleus.

## RESULTS

### PRV infection inhibits SG formation

**Fig 1:**
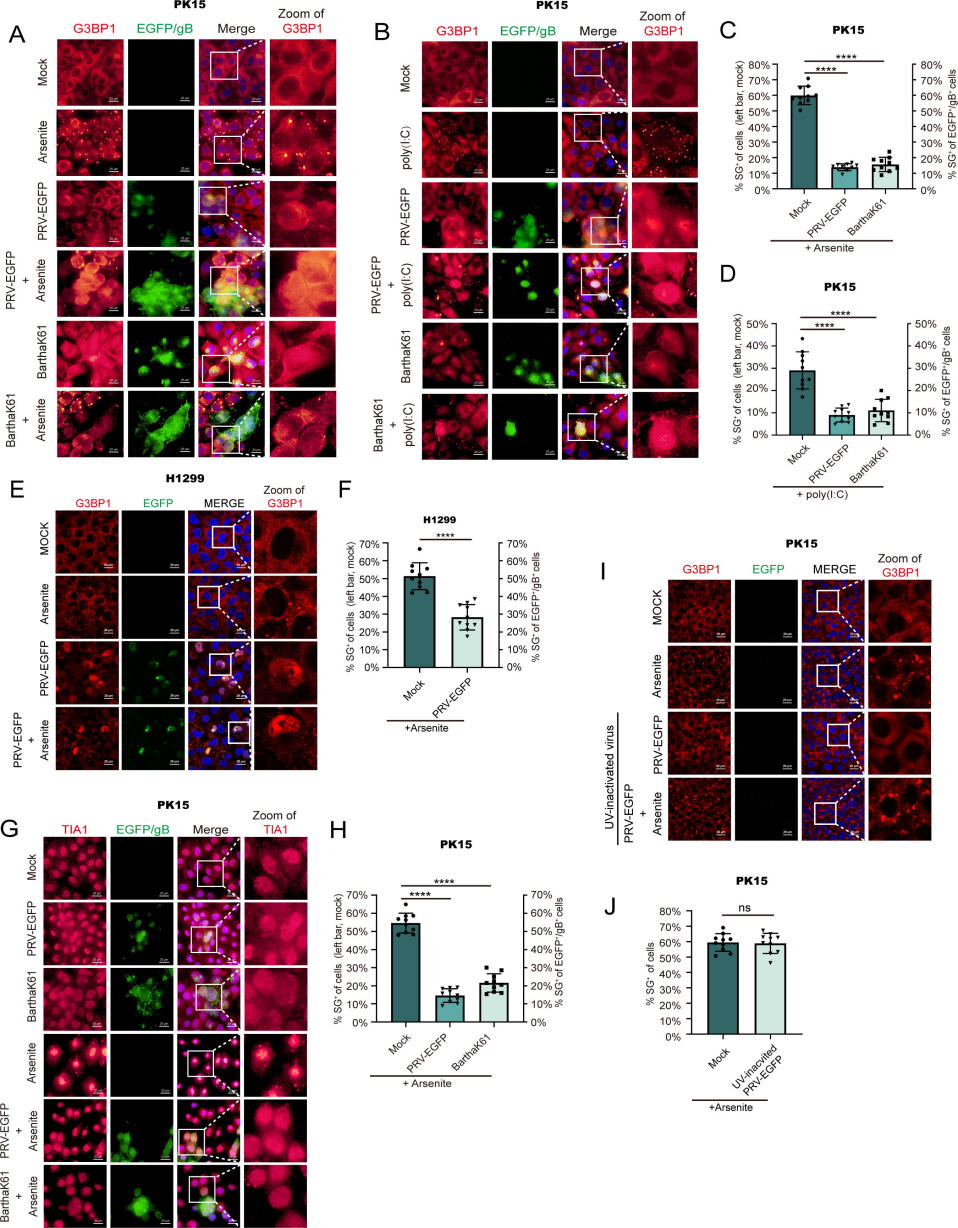
PRV infection inhibits SG formation in host cells. (A, B) PRV-EGFP and BarthaK61 infection inhibit SG formation induced by poly(I:C) or arsenite in PK-15 cells. PK-15 cells were infected with PRV-EGFP or BarthaK61 at 0.1 MOI. SG formation induced by arsenite at 10 hpi (A) or poly(I:C) at 1 hpi. (B) as described in Materials and Methods, cells were fixed at 12 hpi. The virus, SGs, and nuclei were visualized by PRV gB antibody, G3BP1, and DAPI, respectively. The images were acquired with a fluorescence microscope. (G) Cells were treated as described in (A). The cells were fixed at 12 hpi. The virus, SGs, and the nucleus were visualized by anti-PRV-gB antibody, anti-TIA1 antibody, and DAPI. Images were acquired with a fluorescence microscope. (E) PRV-EGFP infection inhibits SG formation induced by arsenite in H1299 cells. H1299 cells were infected with PRV-EGFP. SG formation induced by arsenite at 10 hpi. Cells were fixed at 12 hpi, and the virus, SGs, and nuclei were visualized by PRV gB antibody, G3BP1, and DAPI, respectively. The images were acquired with a confocal microscope. (I) UV-inactivated PRV infection does not inhibit SG formation in PK-15 cells. PRV-EGFP was inactivated by UV for 4 h and infected PK-15 cells with MOI = 1. SG formation was induced by arsenite as described in (A). The images of IFA were acquired with a confocal microscope. (C, D, F, H, J) The percentages of cells or green-fluorescence-positive cells forming stress granules were quantitated in 10 random fields. *P* values were calculated with an unpaired *t*-test (ns, no significant; ****P-value < 0.0001). Scale bars = 20 µm.

Previous studies have shown that PRV infection of host cells inhibits SG formation (32, 33). To test whether this is true for different PRV strains, a recombinant PRV strain derived from the PRV variant HN1201, which stably expresses enhanced green fluorescent protein (EGFP) (34), and a classical PRV vaccine strain BarthaK61 were used to infect PK-15 cells before treating the cells with poly(I:C) or arsenite, which are known triggers for SG formation (21, 35). Cells were stained using an anti-PRV-gB antibody to detect infected cells and an anti-G3BP antibody to visualize SG formation. The microscopy data and statistical analyses showed that both PRV-EGFP and BarthaK61 infection inhibited SG formation triggered by poly(I:C) or arsenite in PK-15 cells (Fig. 1A through D), but UV-inactivated PRV infection failed to suppress SG formation (Fig. 1I and J). As a complementary approach, staining of TIA1, another SG marker, confirmed that PRV infection blocks arsenite-induced SG formation (Fig. 1G and H). PRV infection also inhibits arsenite-induced SG in the H1299 cell line, confirming that similar phenotypes are observed across different cell lines (Fig. 1E and F). Hence, PRV inhibits SG formation in infected host cells.

### PRV infection promotes G3BP1 nuclear translocation in infected host cells

**Fig 2:**
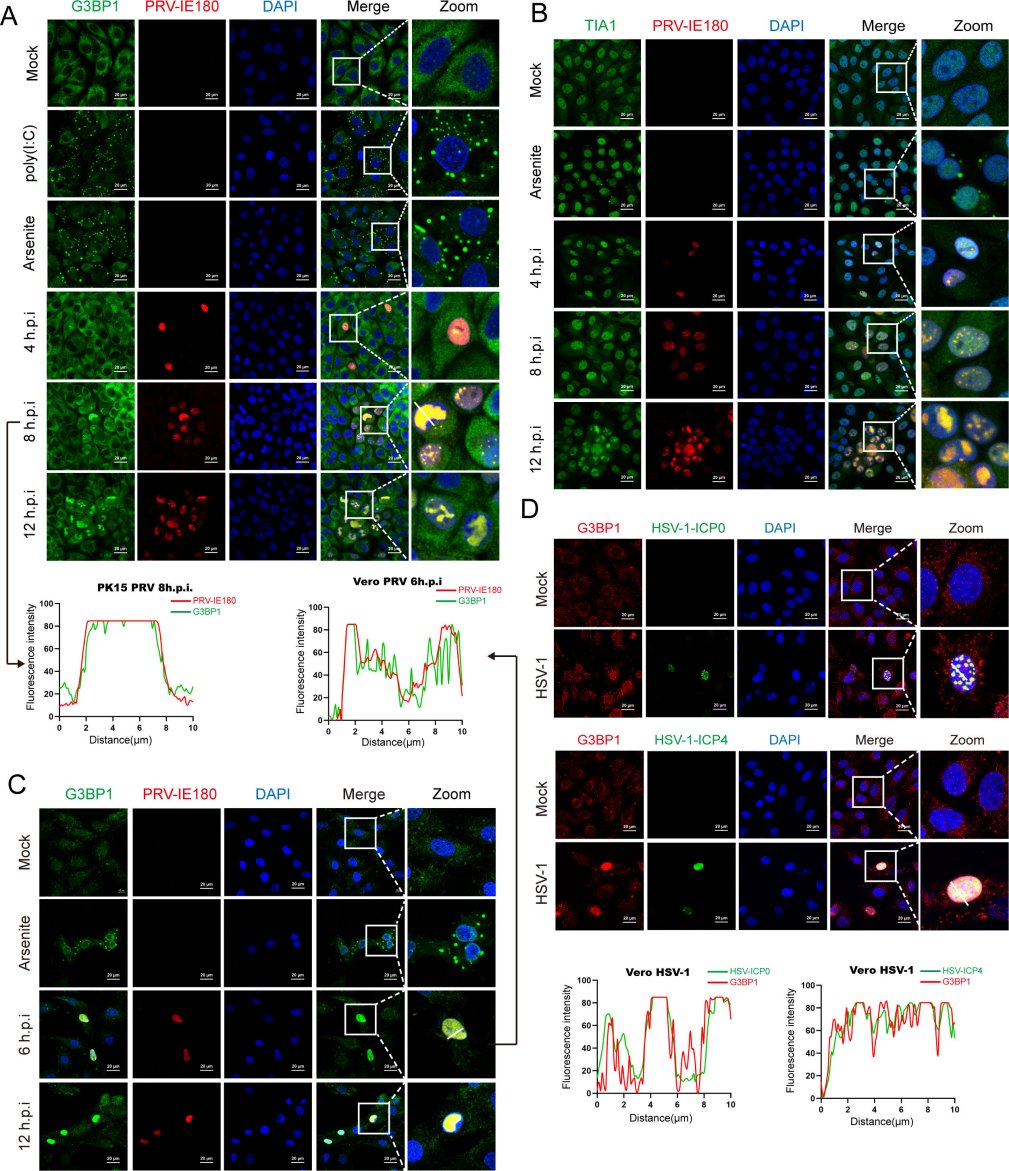
PRV infection does not induce SG formation but promotes G3BP1 nuclear translocation in host cells. (A, B) PK-15 cells were infected with PRV-WT (MOI = 1) for the indicated times. A positive control for SG formation was set by treatment with arsenite or poly(I:C), and mock-infected as a negative control. The cells were stained with anti-G3BP1 (A) or anti-TIA1 (B) antibody for identifying SGs, anti-PRV-IE180 antibody for infected cells. The nuclei were counterstained with DAPI. (C) VeroE6 cells infected with PRV-WT (MOI = 1) for indicated times. A positive control for SG formation was set by arsenite, and mock-infected as a negative control. The virus, SGs, and nuclei were visualized by anti-PRV-IE180 antibody, anti-TIA1 antibody, and DAPI, respectively. (D) VeroE6 cells infected with HSV-1 (MOI = 1) for 12 hpi. SGs and nuclei were visualized by anti-G3BP1 antibody and DAPI, respectively. Anti-HSV-1-ICP0 and anti-HSV-1-ICP4 antibodies were used to visualize the infected cells. Images were acquired with a confocal microscope. The fluorescence colocalization quantification plots were analyzed using ImageJ software. Scale bars = 20 µm.

In order to explore the dynamics of SG formation during PRV infection, we infected PK-15 cells with PRV and observed the formation of SGs at different hours post-infection. Since IE180 protein is the only immediate early protein of PRV, PRV IE180 protein was detected to facilitate the observation of infected cells throughout the infection, and G3BP1 was detected as a marker to evaluate SG formation via immunofluorescence assays. IE180 protein was exclusively expressed in the nucleus from 4 hpi onwards (Fig. 2A). However, we did not observe SG assembly throughout PRV infection. Interestingly, during the course of infection, an increasingly strong colocalization was observed between IE180 and G3BP1 in the nucleus both in PK-15 and Vero E6 cells, while G3BP1 was only observed in the cytoplasm in mock-infected cells (Fig. 2A and C). In line with this, we did not detect TIA1-labeled SGs during PRV infection, and we noticed a colocalization between IE180 and TIA1 in infected cells (Fig. 2B). The results suggest that G3BP1 may translocate from the cytoplasm to the nucleus in PRV-infected cells and may associate with the IE180 protein. To explore if this phenomenon could also be observed with other alphaherpesviruses, ICP0 and ICP4, two immediate early proteins of HSV-1, were detected in HSV-1-infected Vero E6 cells. Similar to PRV IE180, ICP0 and ICP4 of HSV-1 were found to colocalize with G3BP1 in the nucleus of infected cells (Fig. 2D). These results indicate that the nuclear localization of G3BP1 may be a conserved phenomenon in cells infected with alphaherpesviruses.

### The PRV IE180 protein blocks SG formation

**Fig 3:**
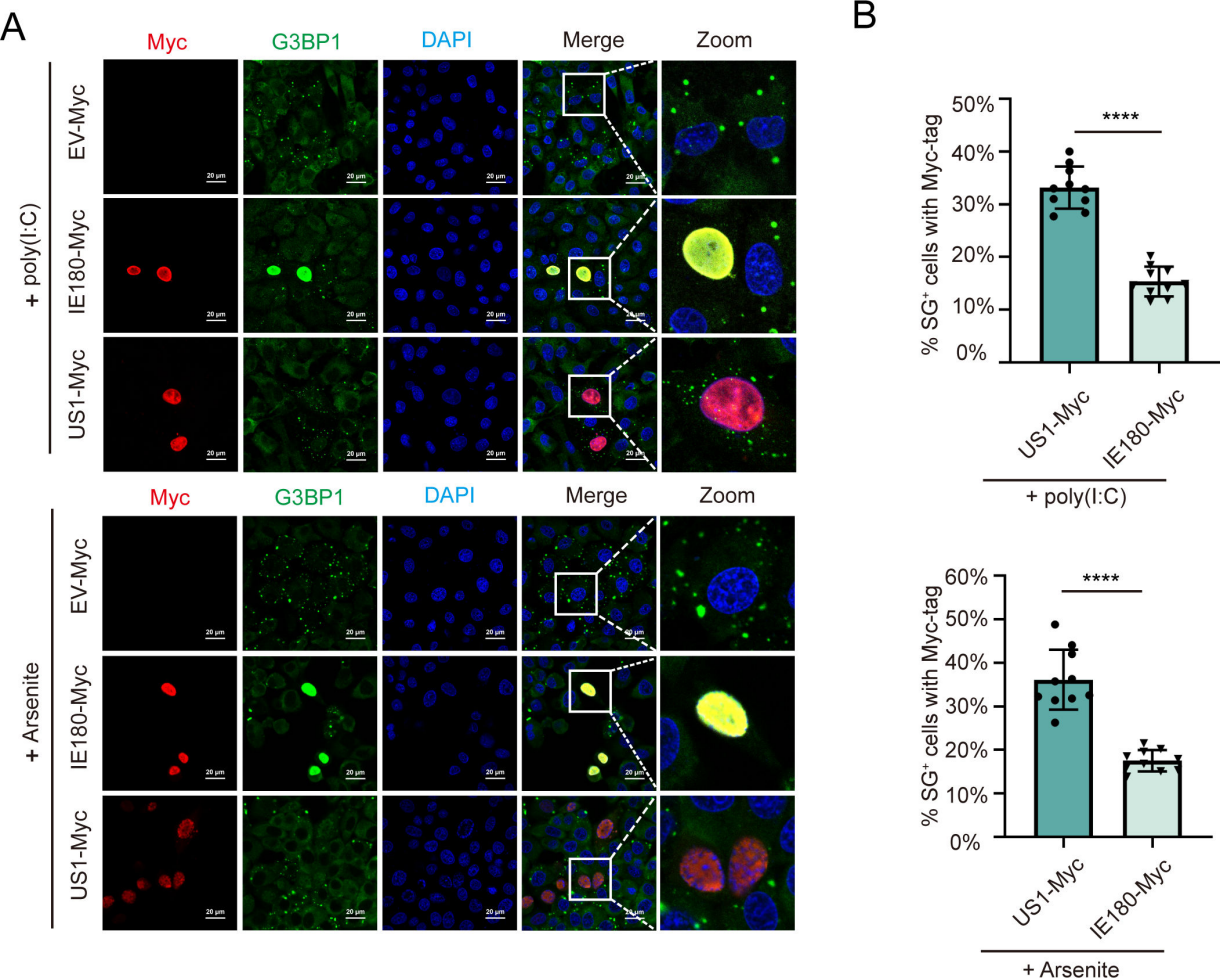
PRV IE180 suppresses SG formation in PK-15 cells. IE180 inhibited SG formation induced by arsenite or poly(I:C). PK-15 cells were transfected with IE180-Myc, or empty vector (EV-Myc) and US1-Myc as negative control. After 24 h post-transfection, cells were treated with poly(I:C) or arsenite to induce SG formation. The cells were fixed at the indicated time, and anti-G3BP1 antibodies were used to monitor SG formation. The nuclei were stained with DAPI. Images were taken using a confocal microscope after immunofluorescence (A). The percentages of SG-positive cells with Myc-tag were quantitated in 10 random fields. P-values were calculated with an unpaired *t*-test (B). (****P-value < 0.0001) Scale bars = 20 µm.

Since PRV suppresses SG formation already early in infection, this suggests that viral protein(s) that are expressed very early in infection may be involved. Hence, we assessed whether transfection of PK-15 cells with the sole immediate-early viral protein of PRV, IE180, or one of the early viral proteins, US1, suppressed SG formation by poly(I:C) or arsenite treatment. Confocal microscopy and statistical analyses of SG formation showed that expression of IE180 significantly inhibited poly(I:C)- or arsenite-induced SG formation as compared with US1 (Fig. 3A and B). Therefore, the above results indicate that expression of the IE180 protein of PRV effectively blocks SG formation.

### PRV IE180 colocalizes and interacts with G3BP1/2 in nucleus

**Fig 4:**
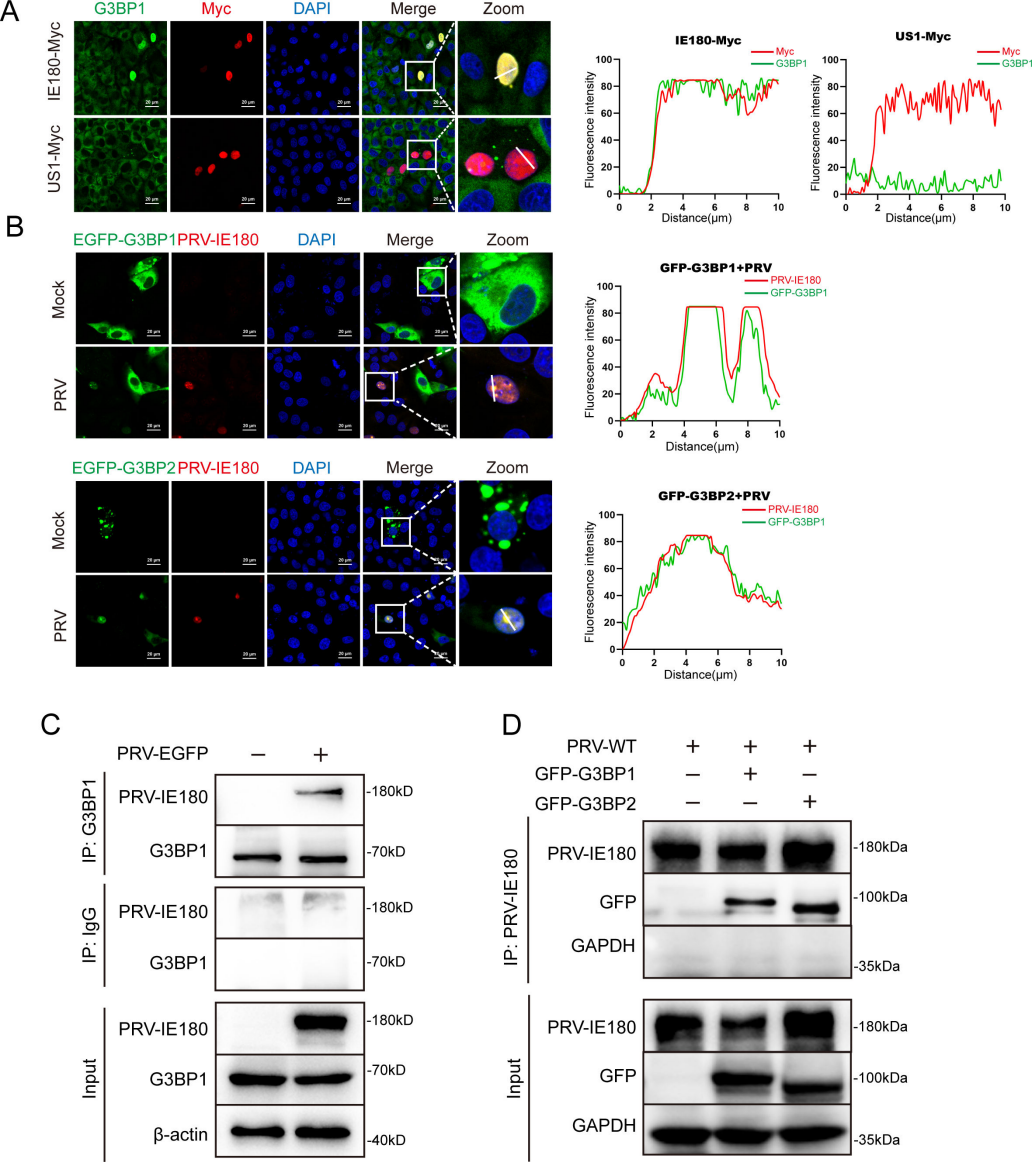
PRV IE180 interacts with G3BP1/2. (A) IE180-Myc colocalizes with endogenous G3BP1 in the nucleus. PK-15 cells were transfected with IE180-Myc, or empty vector (EV-Myc) and US1-Myc as negative control. After 24 h post-transfection, the cells were fixed and stained with anti-G3BP1 and anti-Myc-tag antibodies, and the nuclei were counterstained with DAPI. Images were taken by a confocal microscope after immunofluorescence. (B) PRV IE180 co-located with GFP-G3BP1/2 in nucleus. PK-15 cells were infected with PRV-WT (MOI = 1) for 12 h after being transfected with GFP-G3BP1 or GFP-G3BP2 for 24 h. The cells were stained with anti-PRV-IE180 antibody, and the nuclei were stained with DAPI. Images were taken by a confocal microscope. (C) PRV IE180 interacts with G3BPs in infected PK-15 cells. PK-15 cells were infected with PRV-EGFP (MOI = 1) for 12 h. Cell lysates were immunoprecipitated with anti-G3BP1 antibody and immunoblotted with either anti-IE180 or anti-G3BP1 antibody. (D) PRV IE180 interacts with GFP-G3BP1/2 in PK-15 cells. PK-15 cells transfected with GFP-G3BP1 or GFP-G3BP2 for 24 h, then infected with PRV-WT (MOI = 1) for 12 h, cell lysates were immunoprecipitated with anti-G3BP1 antibody and immunoblotted with either anti-IE180 or anti-GFP-tag antibodies. The fluorescence colocalization quantification plots were analyzed using ImageJ software. Scale bars = 20 µm.

Since we found that overexpressed IE180-myc and G3BP1 colocalize in the nucleus in PK-15 cells in the absence of external stimuli (Fig. 4A), this suggests that IE180 and G3BP1 may interact. Co-immunoprecipitation (co-IP) assays confirmed the interaction between PRV IE180 and endogenous G3BP1 in PRV-infected PK-15 cells (Fig. 4C). G3BP exists as two homologous proteins, G3BP1 and G3BP2 (16). In order to investigate whether both G3BPs translocate into the nucleus during PRV infection, PK-15 cells were infected with PRV upon expression of GFP-G3BP1 or GFP-G3BP2. Immunofluorescence assays showed that both GFP-G3BP1 and GFP-G3BP2 were detected in the nucleus and colocalized with IE180 protein in the nucleus (Fig. 4B). Correspondingly, co-IP assays indicated that PRV IE180 interacts with both GFP-G3BP1 and GFP-G3BP2 (Fig. 4D).

**Fig 5:**
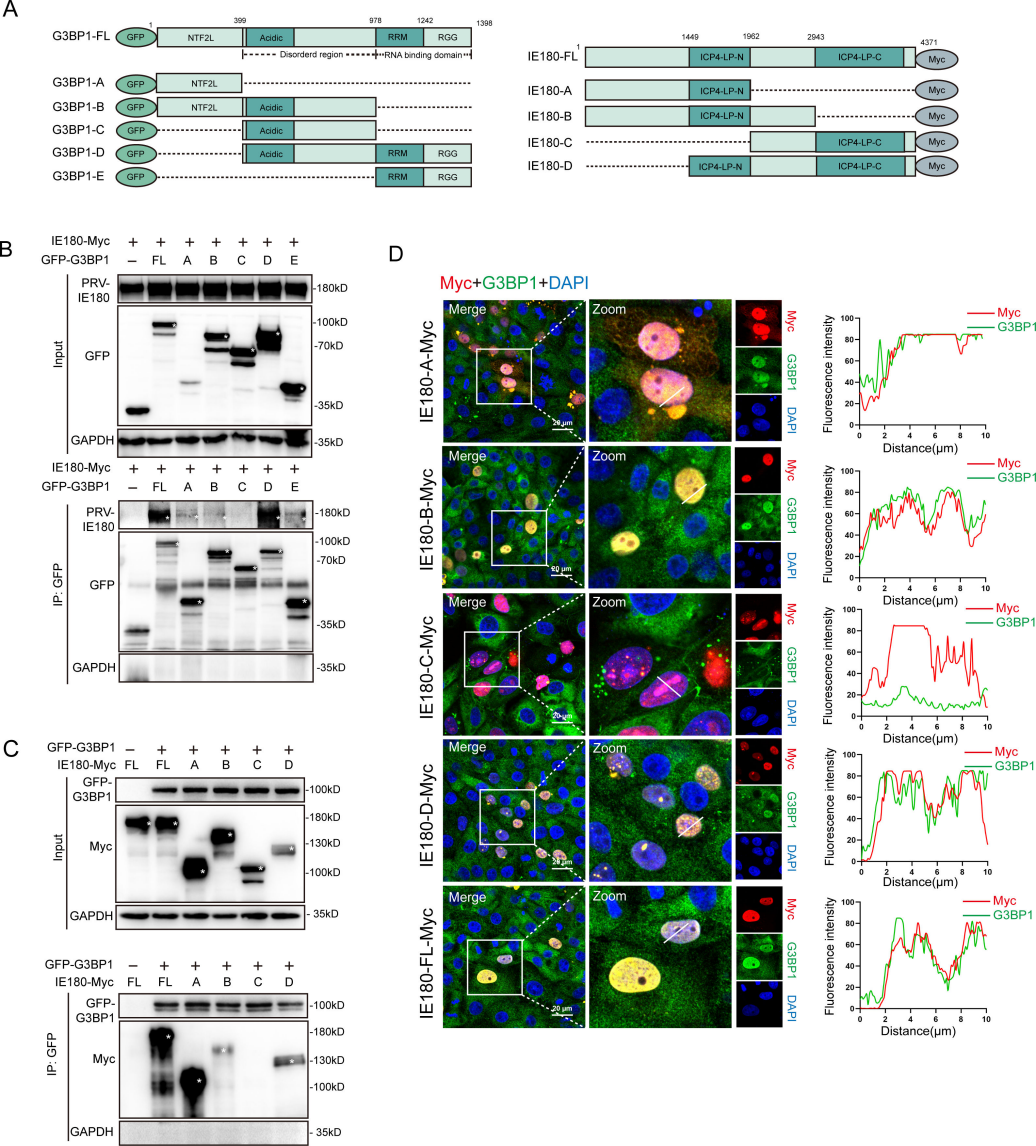
Domains associated with G3BP1 and IE180 interactions. (A) Schematic diagram of the truncated plasmids of GFP-G3BP1 and IE180-Myc. (B) The NTF2L domain and RNA-binding domain of G3BP1 interact with IE180. IE180-Myc co-transfected with full-length (FL) GFP-G3BP1 or truncated plasmids or empty vectors, cell lysates were immunoprecipitated with anti-GFP-tag antibody and immunoblotted with either anti-Myc or anti-GFP antibody. (C) The domain of IE180 interacts with G3BP1. GFP-G3BP1 co-transfected with FL IE180-Myc or truncated plasmids or empty vectors, cell lysates were immunoprecipitated with anti-Myc-tag antibody and immunoblotted with either anti-GFP-tag or anti-Myc-tag antibody. (D) Colocalization analysis of different truncations of IE180 with G3BP1. PK15 cells were transfected with plasmids encoding for various truncations (A–D) or the FL of IE180. After 24 h post-transfection, the images were taken using a confocal microscopy after immunofluorescence. The transfect positive cells, SGs, and nuclei were visualized by anti-Myc-tag antibody, anti-G3BP1 antibody, and DAPI, respectively. The fluorescence colocalization quantification plots were analyzed using ImageJ software. Scale bars = 20 µm.

To delineate the domains involved in the G3BP-IE180 association, plasmids expressing truncated variants of IE180 and G3BP1 were tested in co-IP assays. Since G3BP1 and G3BP2 are homologous proteins, we only constructed truncated variants of G3BP1 (Fig. 5A). Plasmids expressing full-length (FL) or truncated GFP-G3BP1 were co-transfected with IE180-Myc in 293T cells, which were then subjected to co-IP assays. The nuclear transport factor 2-like (NTF2L) domain and RNA binding (RBD, including RRM and RGG) domain of G3BP1 showed an association with IE180-Myc (Fig. 5B). Next, plasmids expressing FL or truncated IE180-Myc were co-transfected with GFP-G3BP1, and cells were again subjected to co-IP assays. The results showed that the ICP4-LP-N domain of IE180 is required for the interaction between IE180 and G3BP1 (Fig. 5C). Consistent with the co-IP results, the immunofluorescence data also indicate that, in contrast to wild type and other truncated variants of IE180, the IE180 truncation lacking the ICP4-LP-N domain (IE180-C) does not colocalize with G3BP1 (Fig. 5D).

These findings suggest that the NTF2L and RBD domains of G3BP1 and the ICP4-LP-N domain of IE180 are of high relevance in the interaction between G3BP1 and IE180.

### IE180 hijacks G3BP into the nucleus to affect SG formation

**Fig 6:**
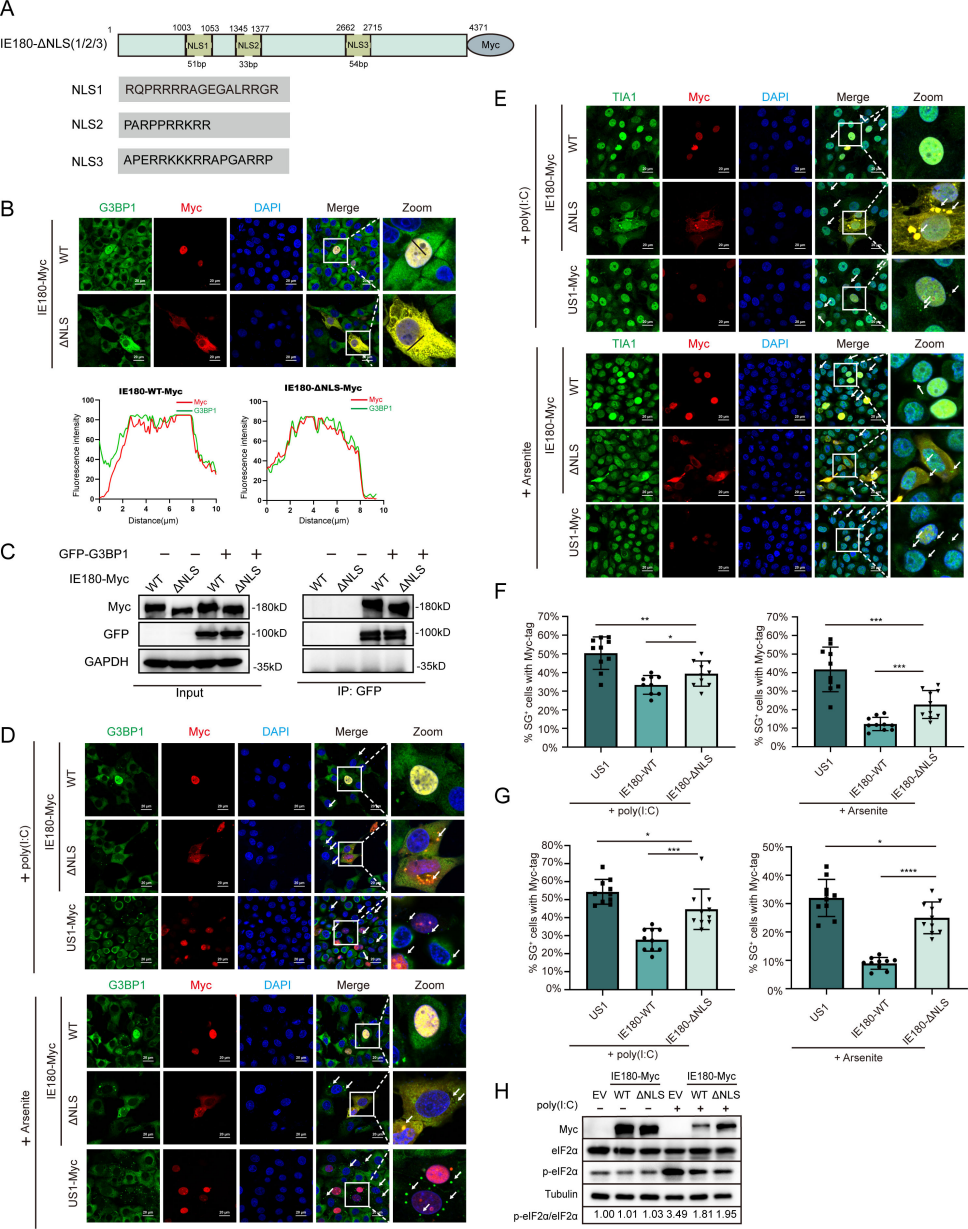
The absence of NLS impairs the ability of IE180 to suppress SG formation. (A) Schematic diagram of IE180-Myc with NLS deficiency. The predicted NLS of IE180 and the missing NLS1/2/3 positions on the IE180-Myc plasmid are shown in the schematic diagram. (B, C) The absence of NLS alters the subcellular localization of IE180, but does not affect the colocalization and interaction between IE180 and G3BP1. (B) PK15 cells were transfected with plasmids of IE180-WT-Myc or IE180-ΔNLS-Myc. After 24 h post-transfection, the images were taken using a confocal microscopy after immunofluorescence. The corresponding proteins were labeled with an anti-Myc-tag antibody and an anti-G3BP1 antibody, respectively, and the nuclei were labeled with DAPI. (C) The indicated plasmids were transfected in 293t cells. After 24 hours post-transfection, immunoprecipitation was performed, and western blot analysis was followed to detect the indicated proteins. (D-E) The capacity of IE180-ΔNLS-Myc to suppress SG formation was inferior to IE180-WT-Myc. PK-15 cells were transfected with IE180-WT-Myc or IE180-ΔNLS-Myc plasmids, US1-Myc as negative control. After 24 h post-transfection, cells were treated with poly (I:C) or arsenite to induce SG formation. The cells were fixed at the indicated time, and images were taken using a confocal microscope after immunofluorescence. Anti-G3BP1 (D) or anti-TIA1 (E) antibodies were used to monitor SG formation. The nuclei were stained with DAPI. (F, G) The percentages of SG-positive cells with Myc-tag were quantitated in 10 random fields. *P* values were calculated with an unpaired *t*-test (****P value < 0.0001, ***P value < 0.001, **P value < 0.01, *P value < 0.05; ns, no significant). Scale bars = 20 µm. (H) The absence of predicted-NLS does not affect the inhibitory effect of IE180 on p-eIF2α. PK15 cells were transfected with plasmids of IE180-WT-Myc, IE180-ΔNLS-Myc, and empty vector control. After 24 h post-transfection, the cells were treated with poly(I:C) as Materials and Methods described, after which Western blot analysis was conducted at the specified time points.

G3BPs are generally present in the cytoplasm to induce SG formation (16). Previous reports suggested that G3BPs shuttle between the cytoplasm and the nucleus upon specific stimulation (36–38). We wonder whether the nuclear localization of IE180 affects the subcellular localization of G3BP. We used NLStradamus software to predict the putative nuclear localization sequence (NLS) of IE180 with a posterior probability greater than 80%. An IE180 expression plasmid lacking the putative NLS (IE180-ΔNLS-Myc) was constructed (Fig. 6A). Although the modified IE180 protein was still expressed in the nucleus in some cells transfected with IE180-ΔNLS-Myc, it was observed to be expressed in the cytoplasm in most cells and still colocalized with G3BP1 (Fig. 6B). Co-IP assays verified that the absence of the NLS in IE180 does not affect its interaction with G3BP1 (Fig. 6C). Hence, the deletion of the NLS altered the subcellular localization of IE180 without impacting its interaction with G3BP1. SG formation induced by poly(I:C) or arsenite was significantly increased in cells transfected with IE180-ΔNLS-Myc compared with IE180-WT-Myc-transfected cells (Fig. 6D and F). To confirm that the observed aggregated G3BP1 foci were bonafide SGs, we also assessed SG formation using the TIA1 marker, which generated similar results (Fig. 6E and G). Nonetheless, the SG formation ratio in IE180-ΔNLS-Myc-expressing cells was still lower than that in US1-Myc-expressing cells, indicating that IE180-ΔNLS-Myc suppresses SG formation but not as efficiently as IE180-WT-Myc. Previous studies have shown that IE180 expression can suppress phosphorylation of eIF2α (31). We found that expression of either IE180-WT or IE180-ΔNLS suppresses the increase in eIF2α phosphorylation levels induced by poly(I:C) (Fig. 6H), which may contribute to the ability of IE180-ΔNLS-Myc to suppress SG formation.

These data suggest that IE180 hijacks G3BP1 into the nucleus by interacting with G3BP1, which contributes to the inhibition of SG assembly.

### PRV infection and IE180 expression inhibit eIF2α-independent SG formation in host cells

**Fig 7:**
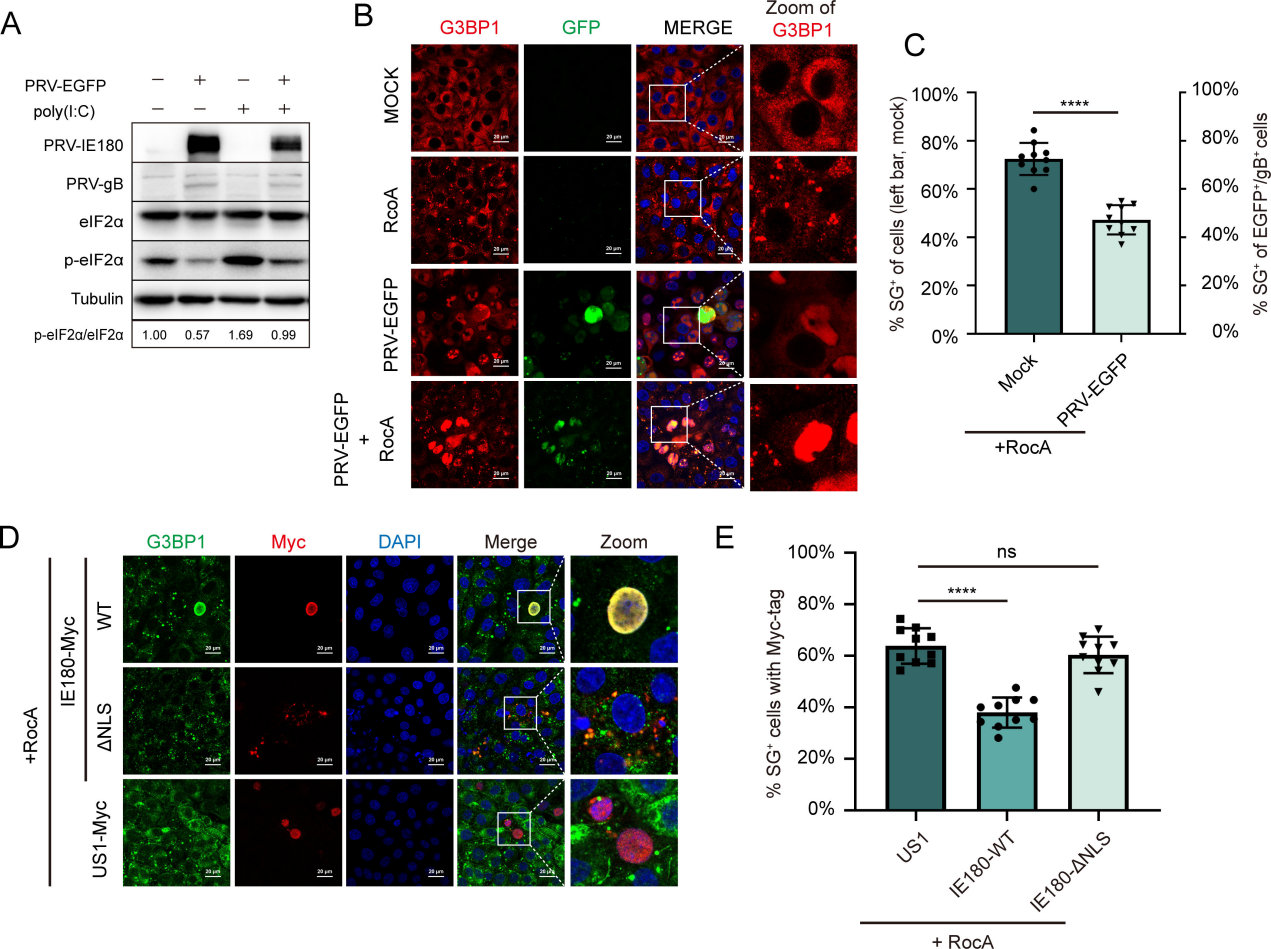
PRV infection or IE180 transfection also inhibits eIF2α-independent SG formation in host cells. (A) PRV infection inhibits the phosphorylation of eIF2α induced by poly (I:C) in PK15 cells. PK15 cells were infected with PRV-EGFP at MOI = 0.1, and poly(I:C) was transfected at 1 hpi. Cells were harvested at 12 hpi. for western blot analysis to detect the levels of indicated proteins. (B) PRV infection inhibits SG formation induced by RocA in PK-15 cells. PK-15 cells were infected with PRV-EGFP at 0.1 MOI. SG formation induced by RcoA at 8 hpi. as described in Materials and Methods, cells were fixed at 12 hpi. The virus, SGs, and nuclei were visualized by anti-PRV IE180 antibody, anti-G3BP1 antibody, and DAPI, respectively. Images taken using a confocal microscope after immunofluorescence. (C) The percentages of SG-positive cells or green-fluorescence-positive cells were quantitated in 10 random fields. (D) PK-15 cells were transfected with IE180-WT-Myc or IE180-ΔNLS-Myc plasmids, US1-Myc as negative control. Cells were treated with 3 µM RocA for 4 h to induce SG formation after 24 h post-transfection. The cells were fixed at the indicated time, and images were taken using a confocal microscope after immunofluorescence. Indicator protein labeling, the statistical methods for SG formation were the same as in Fig. 6D and F. (E) The percentages of SG-positive cells with Myc-tag were quantitated in 10 random fields. *P* values were calculated with an unpaired *t*-test (****P value < 0.0001; ns, no significant). Scale bars = 20 µm.

Poly(I:C) and arsenite both induce SG formation through the phosphorylation of eIF2α, with elevated levels of phosphorylated eIF2α (p-eIF2α) being one of the significant triggers for SG formation (16). PRV infection suppresses the increase in p-eIF2α levels induced by poly(I:C) (Fig. 7A), which is consistent with reported research results (31, 32). However, our study reveals that PRV may also inhibit SG formation through a mechanism independent of eIF2α regulation. To further investigate the effect of PRV or IE180 on SG formation independent of eIF2α, we utilized Rocaglamide (RocA), a compound known to induce SGs by targeting the eIF4E complex in an eIF2α-independent manner (39), and evaluated the effects of PRV on SG formation induced by RocA (Fig. 7B and C). The data show that PRV infection also suppresses SG formation triggered by RocA. The effect of expression of IE180 or NLS-deficient IE180 on RocA-induced SG formation was also investigated (Fig. 7D and E). Compared to US1-Myc, SG formation was significantly suppressed in cells transfected with IE180-WT-Myc, while no significant change was observed in cells transfected with IE180-ΔNLS-Myc. These results demonstrate that both PRV infection and IE180 expression inhibit eIF2α-independent SG formation, whereas IE180 loses this ability when the predicted-NLS is deleted. This finding supports the notion that IE180 expression and PRV infection can suppress SG formation via pathways that do not involve eIF2α.

### G3BP and SG formation impair PRV replication in host cells

**Fig 8:**
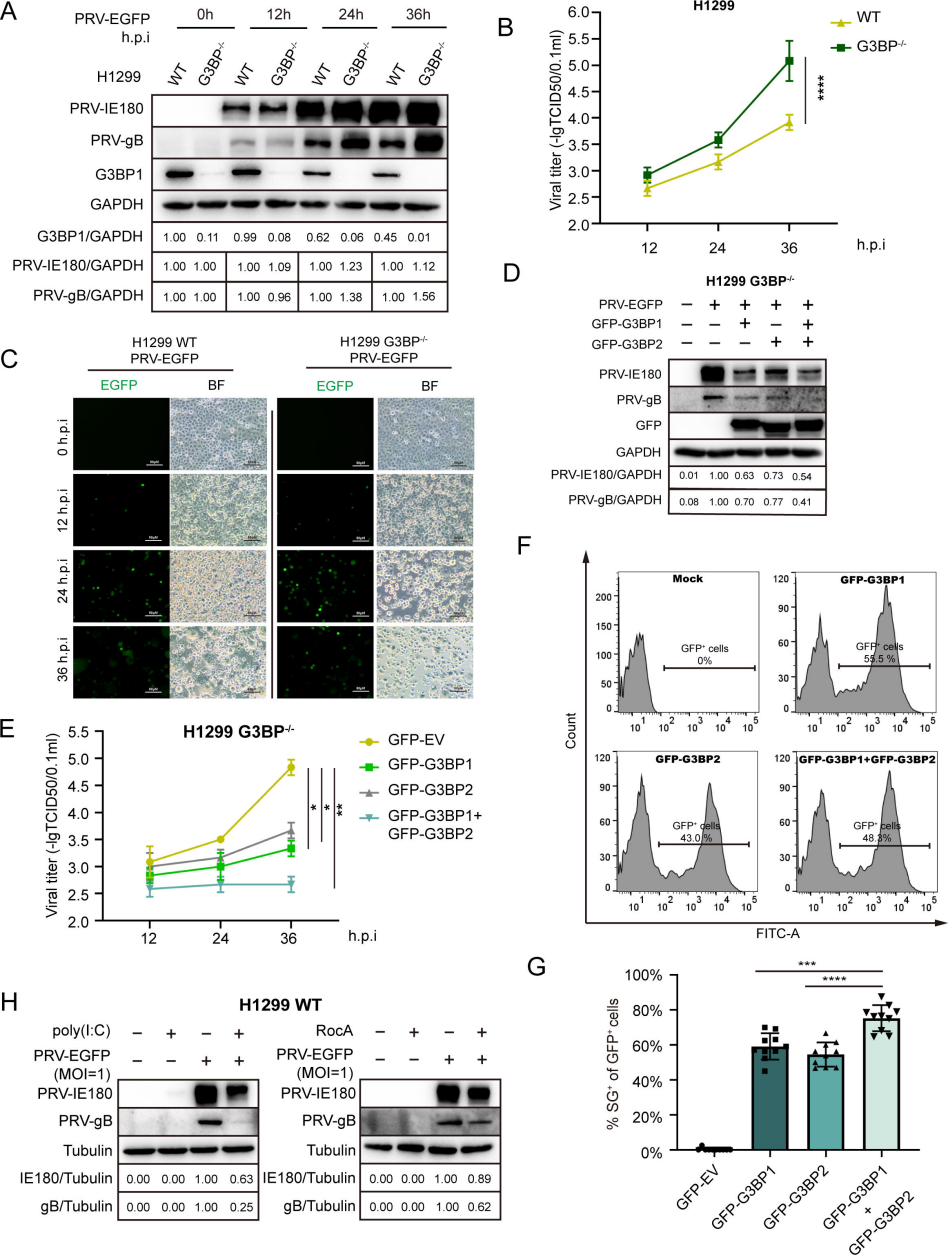
G3BPs and SG formation in host cells inhibit replication of PRV. (A–C) G3BPs restricted the replication of PRV. H1299 WT or H1299 G3BP1^−/−^ cells were infected with PRV-EGFP. Cells were harvested at different times and subjected to western blot analysis (A). Cell culture supernatants were collected at the indicated times, and titers were determined using the TCID_50_ assay (B). Images taken using a fluorescence microscope showed PRV-infected cells based on EGFP and light microscope exhibition cell morphology of H1299 WT or H1299 G3BP1^−/−^ cells (C). Scale bars = 50 µm. (D, E) Overexpression of G3BP1 or G3BP2 inhibits PRV replication in H1299 G3BP1^−/−^ cells. H1299 G3BP1^−/−^ cells were infected with PRV-EGFP (MOI = 1) for 12 h after being transfected with GFP-EV, GFP-G3BP1, or (and) GFP-G3BP2 for 24 h. Western blot analysis (D) and virus titration (E) in the cell culture supernatants were performed at indicated times. (F) The transfection efficiency of GFP-G3BP1/2 was measured by flow cytometry. H1299 G3BP1^−/−^ cells were transfected with GFP-G3BP1 or (and) GFP-G3BP2 for 24 h, respectively, with untreated cells as negative control. Analyze the data using FlowJo software. (G) The SG formation rate of GFP-positive cells was quantitated in 10 random fields. Anti-TIA1 antibody was used to monitor SG formation. *P* values were calculated with an unpaired *t*-test. (H) Poly (I:C) and RocA-induced SG formation are detrimental to PRV replication. H1299 cells were pretreated with 1.5 µg/mL poly (I:C) for 12 h and 3 µM RocA for 4 h before infecting with PRV-EGFP (MOI = 1). Then, the treatments were maintained throughout the infection period, using a low concentration (1 µM) of RocA to ensure cell viability. Western blot analysis at 12 hpi. Graphs (B and F) represent independent biological replicate samples (*N* = 3). Error bars = standard deviation. Two-way ANOVA and Dunnett’s multiple comparison tests (****P value < 0.0001, ***P value < 0.001, ** *P* value < 0.01, *P value < 0.05).

To further investigate the role of G3BP in PRV infection, we used an H1299 G3BP knockout (H1299 G3BP1^−/−^) cell line (40). The expression of G3BP1 in H1299 G3BP1^−/−^ cells was severely reduced, and we observed that viral gB protein expression levels were substantially increased in PRV-infected H1299 G3BP1^−/−^ cells compared to parental wild-type (WT) cells (Fig. 8A). Consistently, the viral titer in PRV-infected H1299 G3BP1^−/−^ cells was significantly higher than that in H1299 WT cells (Fig. 8B). In line with this, the intrinsic GFP signal of the used PRV strain appeared to be increased in G3BP1^−/−^ H1299 cells compared to that in WT cells (Fig. 8C). Levels of viral proteins and viral titers were significantly suppressed by overexpression of G3BP1 or (and) G3BP2 in H1299 G3BP1^−/−^ cells (Fig. 8D and E). This indicates that PRV replication is inhibited by the expression of G3BP1/2, with co-transfection of GFP-G3BP1/2 yielding a more pronounced antiviral effect. Flow cytometry showed similar transfection efficiencies upon GFP-G3BP1 and/or GFP-G3BP2 transfection (Fig. 8F). Given that overexpression of G3BP1 or G3BP2 was reported to induce SG formation (41), we also quantified the SG formation rate in transfected cells (Fig. 8G). Results in Fig. 8G showed that co-transfection of GFP-G3BP1/2 led to more robust SG formation despite stable transfection efficiency. This indicates that SG formation may contribute to the observed inhibition of PRV replication in Fig. 8D and E. However, whereas transfection efficiencies in G3BP overexpression assays reached around 50% (Fig. 8F), the suppressive impact of G3BP overexpression on virus titers was 10- to 100-fold (Fig. 8E). This suggests that G3BP overexpression in transfected cells may also affect virus replication in non-transfected cells. This would be in line with several reports linking G3BP (over)expression and G3BP-induced SG formation to the production of type I interferon, including in PK15 cells (35, 42–46).

To further determine the impact of SG formation on PRV replication, we induced SG formation in H1299 cells using poly(I:C) and RocA, followed by PRV infection. PRV-IE180 and PRV-gB protein levels decreased upon either poly (I:C) or RocA treatment, indicating that SG formation alone is sufficient to suppress PRV infection and replication (Fig. 8H).

In summary, our results show that G3BP protein expression and SG formation in host cells are detrimental factors for PRV replication.

## DISCUSSION

For decades, PRV has been responsible for significant economic losses in the worldwide swine industry, more recently especially through the emergence of mutant strains in Asia (1, 3). The report of rare potential human cases of PRV infection in Asia in recent years has further increased its importance in the field of public health (4). In this study, we first verified the suppression of SG formation by different strains of PRV and discovered that the immediate early protein (IE180) suppresses SG formation. Subsequently, a novel mechanism to block SG formation in response to virus infection was revealed. G3BP proteins were translocated in the nucleus during PRV infection as soon as IE180 was expressed. In line with this, we found that IE180 and G3BP interact, and critical protein domains involved in this interaction were identified. We further showed that the nuclear translocation of G3BP1 was a disadvantage for SG formation. This discovery provides a new perspective on the evasion of the intrinsic antiviral host cell defense mechanism by PRV.

As a hub of cell translation and innate immunity, SGs generally occupy a decisive standing in the antiviral response of eukaryotic cells (47). In addition to the eIF2α-dependent and eIF4A-related pathways of SG formation, other stressors can directly alter the intracellular environment to induce phase separation (14). Following the imposition of stress, SG assembly is catalyzed by the cumulative effect of many weak, multivalent interactions among the newly available mRNAs with RNA-binding proteins (48). A certain threshold concentration of G3BP1/2 proteins is critical for SG formation (16). Though several studies have shown that PRV infection suppresses eIF2α-dependent SG formation, viral proteins involved in the suppression of SG formation have not been reported (32, 33, 49). Our study showed that different strains of PRV inhibit SG formation induced by poly(I:C) or arsenite in the early stage of infection, and that expression of the viral IE180 protein is sufficient to suppress SG formation.

G3BP1 is a central node of the protein-RNA interaction network in SG formation, which functions as a molecular switch that triggers LLPS in response to the increase of intracellular free RNA concentrations (16). In previous studies, G3BP1 was found to act as an anchor for viral proteins to suppress SG formation (21, 22, 50). Notably, G3BP2 is a homologous protein of G3BP1 and is also pivotal in SG assembly. G3BP proteins are generally expressed in the cytoplasm to induce SG assembly (16, 51). However, both G3BP proteins were observed in the nucleus during PRV infection. Several studies reported nuclear localization of G3BP1 during pathological development of disease or virus infection (36, 52). However, the effect of changes in subcellular localization of G3BP1 on SG formation has yet to be addressed. Here, we propose the hypothesis that the nuclear transfer of G3BP1 was unfavorable for SG formation, which is supported by the results in Fig. 6. Moreover, our results indicate that nuclear translocation of G3BP proteins was caused by their interaction with IE180, which suggests that IE180 hijacks G3BP proteins into the nucleus, a mechanism of interference with SG formation that, to the best of our knowledge, has not been reported before in viral infections. G3BP1 is known to shuttle between the nucleus and the cytoplasm (53). Hence, it is also possible that IE180 retains G3BP1 in the nucleus and prevents its export to the cytoplasm rather than translocating cytoplasmic G3BP1 to the nucleus. Further research will be needed to discriminate between these possibilities and to dissect the exact mechanism of how IE180 affects subcellular localization.

The absence of the IE180 gene renders PRV non-replicative (30), which limits our ability to experimentally assess the contribution of IE180 in the inhibition of SG formation during PRV infection. Indeed, since the absence of IE180 results in a lack of expression of early and late viral genes in PRV-infected cells (30), the use of an IE180 null PRV strain does not allow us to discriminate between the role of IE180 and that of other viral proteins. However, we have predicted the NLS of IE180 and constructed an expression plasmid for an IE180 mutant lacking the NLS. We were unable to completely alter the localization of IE180 upon mutation of the predicted NLS, which is consistent with previous studies (54). Nonetheless, the partial relocation of IE180 to the cytoplasm upon mutation of the predicted NLS, which was accompanied by a reduced ability of IE180 to inhibit SG formation, supports our hypothesis that the inhibitory effect of IE180 on SG formation may at least partly be due to nuclear retention of the G3BP proteins with which it interacts. We observed that a truncated version of IE180 lacking NLS3 (IE180-A) exhibited a more pronounced tendency to be expressed in the cytoplasm compared to truncations lacking NLS1/2 (IE180-B, IE180-C, IE180-D) (Fig. 5D). This suggests that NLS3 may play a more significant role than NLS1/2 in its nuclear localization. In addition, the IE180-C truncation does not interact with G3BP1, and G3BP1 failed to translocate to the nucleus in IE180-C expressing cells.

Interestingly, we also observed that immediate early proteins of HSV-1 (ICP0, ICP4) colocalize with G3BP proteins in the nucleus during HSV-1 infection, suggesting that this phenomenon may be conserved among alphaherpesviruses. Further, at late stages of infection in PRV-infected PK15 and Vero cells, G3BP1, TIA1, and IE180 localized in subnuclear regions that are devoid of marginalized host chromatin, which typically corresponds with viral DNA replication compartments (Fig. 2). Additional research is needed to confirm this potential colocalization with viral replication compartments and to assess the functional and biological consequences of such particular localization.

G3BP1 and G3BP2 exhibit a similar structure, including a nuclear transport factor 2-like (NTF2L) domain at the N-terminus, an acidic and proline-rich region (PxxP), an RNA recognition motif (RRM), and a C-terminal arginine and glycine-rich (RGG) region (55). In this study, by comparing the common structural domains contained in the G3BP1 truncations that interact with IE180, we found that the NTF2L and RRM/RGG domains of G3BP are of particular importance in the interaction with the IE180 protein. It is worth noting that the negative results of G3BP1-C or IE180-C in Fig. 4B or 4C may not be due to the absence of key interaction domains, but rather a result of misfolding of the truncated plasmids. Both NTF2L and RRM/RGG domains are crucial for SG aggregation (16). In addition, the NTF2L domain contributes to the dimerization of G3BP proteins, while the RRM and RGG regions both belong to the RNA-binding domain of G3BP proteins and are associated with RNA binding (56). The RGG region has also been implicated in the nucleo-cytoplasmic shuttling of G3BP1 (53).

It is noteworthy that some viruses facilitate replication by manipulating SG formation through more than one pathway. For example, on the one hand, SARS-CoV-2 blocks activation of the PKR-mediated integrated stress response and subsequent SG formation through the N2b structural domain of the N protein (57). On the other hand, SARS-CoV-2 infected cells also display reduced levels of G3BP1 protein that colocalize in the nucleus with nucleolin, which may also impair SG formation (52). In herpesviruses, HSV-1 can inhibit SG formation through the viral ICP0 protein, which inhibits the activation of PERK during the early stages of viral infection. In addition, the viral gB protein of HSV-1 also regulates UPR responses by interacting with PERK, which possibly may affect SG formation (58). The results in Fig. 6 imply that PRV IE180 may inhibit SG formation in more than one way. Our data in Fig. 6H indicate that IE180 can suppress SG formation by inhibiting the phosphorylation of eIF2α, in line with previous research (31). The absence of the predicted NLS did not affect this inhibitory effect of IE180, which provides a potential explanation for why IE180-ΔNLS still possesses some capacity to inhibit SG formation. In addition, the interaction between IE180 and G3BP1 may prevent G3BP1 from inducing SG formation, which could also be one of the reasons why IE180 retains some capacity to inhibit SG formation even after the deletion of the NLS.

**Fig 9:**
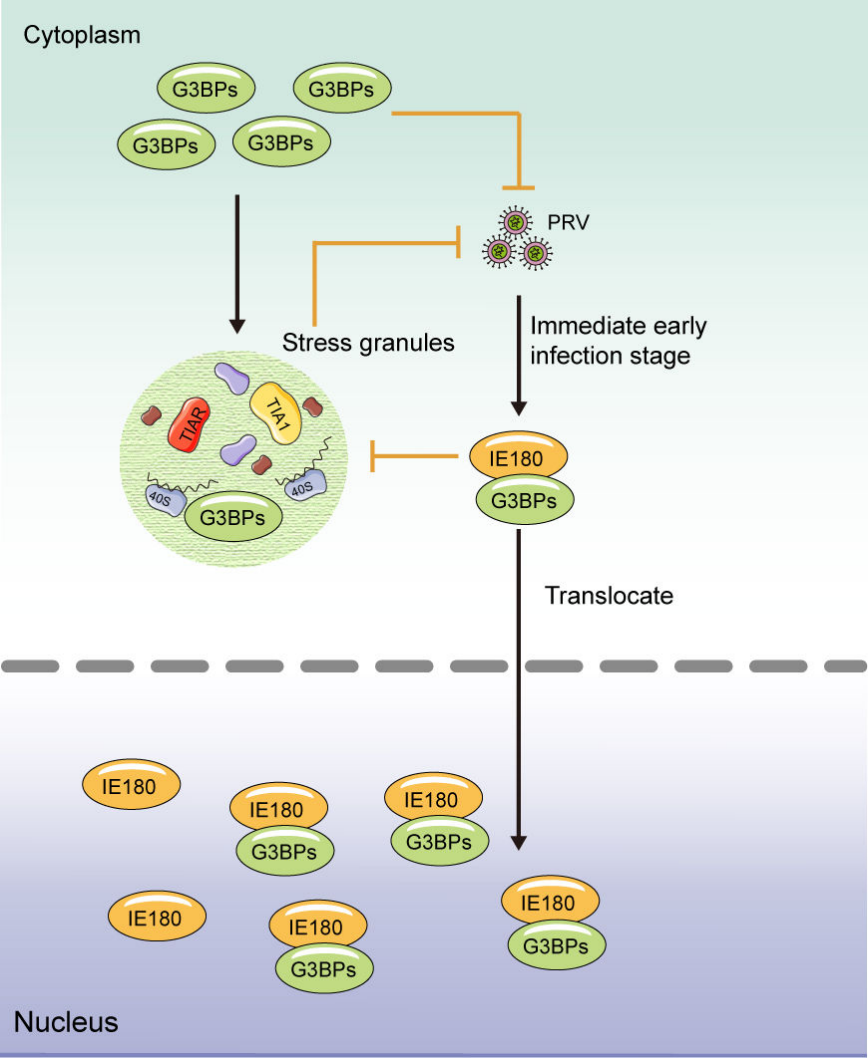
Model of PRV IE180 protein hijacks G3BPs into the nucleus to inhibit SG formation. G3BP1 is crucial for SG assembly as a tunable switch. In the immediate early stage of PRV infection, PRV IE180 interacts with G3BPs and hijacks them into the nucleus to impair SG assembly. Meanwhile, G3BPs and SGs in host cells are adverse to viral replication. The black lines and orange blunt-ended lines indicate activation and inhibition, respectively.

In conclusion, our study identified that the viral IE180 protein of PRV is able to inhibit SG formation (Fig. 9). We demonstrated that IE180 hijacks G3BP proteins into the nucleus to prevent SG assembly, which represents a novel viral strategy to suppress SG assembly.

## MATERIALS AND METHODS

### Cells and viruses

PK-15 cells, Vero-E6 cells, and HEK293 T cells were stored in our lab. H1299 WT or H1299 G3BP1^−/−^ cells were generously given by Prof. Chan Ding (Chinese Academy of Agricultural Sciences) and have been described previously (40). A recombinant virus rPRV HN1201-EGFP-Luc (PRV-EGFP) was a generous gift from Prof. Beibei Chu (Henan Agricultural University) and have been described previously (34). The WT PRV JS-2020 strain (PRV-WT) and BarthaK61 vaccine strains were stored in our laboratory.

### Cell culture, medicine treatment, and virus infection

Cells were cultured in Dulbecco’s modified Eagle’s medium (DMEM, Gibco) supplemented with 10% fetal bovine serum (FBS) and 1% Penicillin-Streptomycin Solution (PS, 100×, NCM Biotech) (Complete medium). All viral stocks were titrated on monolayers of PK-15 cells.

Cells were treated with 0.5 µM arsenite (Sigma, S7400) for 1 h, or 1.5 µg/mL poly(I:C) (Invitrogen, tlrl-pic) for 12 h, or 3 µM RocA (MCE, HY-19356) for 4 h to induce SG formation. Arsenite and RocA were pre-added in FBS-free DMEM with homogeneous mixing, and then the mixed liquid was added to PK-15 cells in the dishes. Poly(I:C) treatment was the same as transfection with Lipofectamine 2000 (Thermo Fisher).

Cells were infected with virus in FBS-free DMEM for 1 h, then maintained in DMEM with 1% FBS and 1% PS (Maintenance medium).

### Plasmid construction and transfection

G3BP1/2 and mutants of G3BP1 were expressed by cloning the indicated gene into pEGFP-N1 plasmids. IE180, IE180-ΔNLS, and mutants of IE180 were expressed by cloning the indicated gene into pcDNA3.1-Myc-C plasmids. The NLS sequence of IE180 was predicted by NLStradmus (http://www.moseslab.csb.utoronto.ca/NLStradamus/). Plasmids were transfected with Lipofectamine 3000 (Thermo Fisher) in HEK293T cells and with Hieff *Trans* PEI transfection reagent in PK-15 cells. Detailed transfection methods were described in the instructions.

### Tissue culture infectious dose 50 (TCID_50_)

PRV-EGFP released to the supernatant in the infected PK15 cells and H1299 WT or H1299 G3BP1^−/−^ cells were determined by TCID50 assay. Briefly, the supernatant of infected cells was limitingly diluted in 9× volume of serum-free medium before being added into PK15 cells in 96-well plates for 1 h. Then, the cells were washed with PBS once, and a maintenance medium was added to the wells. Infected cells were observed at 72 hpi based on EGFP.

### Indirect immunofluorescence assay

Cells were seeded in a 12-well plate pre-laid with microscope cover glasses. After the indicated treatment, cells were washed with 1× PBS and immobilized in 1 mL of 4% paraformaldehyde per well at room temperature (RT) for 10 min. Then, the cells were permeabilized with 500 µL of 0.5% Triton-X 100 per well at RT for 10 min. Before incubating primary antibodies, the cells were blocked with 3% bovine serum albumin (BSA) at RT for 1 h. The cells were washed three times with 1× PBS before each step. A total of 300 µL of primary antibody diluted in 3% BSA was incubated with cells at 4°C overnight. After being washed with 0.1% PBST (Tween 20 in PBS) three times, the cells were incubated with 300 µL of secondary antibody diluted in 3% BSA at RT for 1.5 h. Next, the cells were washed with 0.1% PBST three times, and the cells were incubated with 300 µL DAPI (Thermo, D1306, 1:2,000 dilution) at RT for 8 min. The cells with cover glasses were enclosed on slides using Antifade Mounting Medium (Beyotiome, P0126). The cells are observed using a fluorescence microscope or confocal microscopy after being dried at RT.

### Western blot analysis

Cells were lysed in ice-cold RIPA Lysis Buffer (Beyotime, P0013B) with phosphatase inhibitor (Cowin Biotech, CW2383S) for 30 min on ice, with multiple vortex oscillations of the mixture during the lysing period. Cell lysate was centrifuged for 10 min 12,000 × *g* at 4°C. Supernatants were harvested, and protein concentrations were determined by the Pierce BCA protein assay kit (Thermo Fisher, VH312653). Clear cell lysate denaturation with 1× protein loading buffer for 10 min at 100°C. A total of 30–40 μg protein samples of each and protein markers (Vazyme, MP102-01) were separated by 10% or 8% SDS-PAGE gel in 1× SDS PAGE running buffer. The proteins were then blotted onto 0.22 µm PVDF membranes by wet electrophoretic transfer or semi-dry transfer in 1× membrane transfer buffer. Membranes were washed in TBST (1% Tween 20 diluted in TBS) for one time. After blocking in blocking buffer (5% fat-free milk diluted in TBST, or 3% BSA diluted in TBST) at RT for 1 h, membrane incubation with primary antibodies diluted in Universal Antibody Diluent (NCM, WB500D) as indicated in Table 1 overnight (12–18 h), and then incubated with corresponding secondary antibodies diluted in blocking buffer at RT for 1 h. Membranes were washed by TBST three times in a shaker after each incubation was finished. The signal was detected using an enhanced chemiluminescence system with Chemiluminescence Imaging System (Tanon-5200 Multi).

**TABLE 1:** Antibodies

Name of antibody	Application	Source and catalog no.
β-Actin	Western blot 1:10,000	Proteintech, 81115-1-RR
GAPDH	Western blot 1:5000	Proteintech, 60004-1-Ig
G3BP1	Western blot 1:1000	Abcam, ab181150
	Immunofluorescence 1:500	
TIA1	Immunofluorescence 1:200	Proteintech, 12133-2-AP
PRV-gB	Western blot 1:2,000	Given by Prof. Beibei Chu
	Immunofluorescence 1:1,000	
PRV-IE180	Western blot 1:2,000	Laboratory stored
	Immunofluorescence 1:1,000	
HSV-1 ICP0	Immunofluorescence 1:500	Santa Cruz, sc-53070
HSV-1 ICP4	Immunofluorescence 1:500	Santa Cruz, sc-69809
Myc-tag	Western blot 1:2,000	CST, #2276
	Immunofluorescence 1:500	
GFP-tag	Western blot 1:2,000	Proteintech, 50430-2-AP
p-eIF2α	Western blot 1:1,000	CST, 5324T
eIF2α	Western blot 1:2,000	CST, 9721S

### Co-IP assay

Ectopic proteins were expressed in HEK293T cells by transfection of indicated plasmids, and endogenous virus protein was obtained by infected PK-15 cells. Cells were harvested at 24 h post-transfection or 8 h post-infection. Then, the cells were lysed in lysis for IP (Beyotime, P0013J) with PMSF (Beyotime, ST506) for 30 min on ice after being washed by PBS for once. Lysates were centrifuged at 12,000 × *g* for 10 min at 4°C. A total of 50 µL of each supernatant was separated and denatured with 1× protein loading buffer as control, and 200 µL of each was mixed with indicated antibody or IgG of corresponding species overnight (12–18 h). Magnetic beads (Invitrogen, 10004D) were washed using PBS, and then the reaction environment was balanced with IP lysate. The beads were added to co-immunoprecipitated complexes and incubated for 3–4 h at 4°C followed by washing with PBST (0.05% Tween 20) five times. Co-immunoprecipitated complexes were eluted by 1× SDS loading buffer in 100°C laboratory water bath.

### Flow cytometry analysis

After transfected with plasmids for 24 h, G3BP^−/−^ H1299 cells were collected into tubes after digestion with trypsin. Centrifuge the cells at 1,000 × *g* for 5 min and discard the supernatant. Wash cells with 1× PBS by resuspending them in 1 mL of 1× PBS and centrifuge again at 1,000 × *g* for 5 min, and resuspend the pellet in 500 µL of 1× PBS. Cells were collected to detect the GFP fluorescence intensity by flow cytometry (Becton-Dickinson, LSRFortessa).

## Data Availability

All relevant data are within the article.
